# Hospital discharge diagnostic and procedure codes for upper gastro-intestinal cancer: how accurate are they?

**DOI:** 10.1186/1472-6963-12-331

**Published:** 2012-09-21

**Authors:** Efty Stavrou, Nicole Pesa, Sallie-Anne Pearson

**Affiliations:** 1 Adult Cancer Program, Prince of Wales Clinical School, Lowy Cancer Research Centre, University of New South Wales, Sydney, NSW, Australia; 2 Faculty of Pharmacy, The University of Sydney, Sydney, NSW, Australia

**Keywords:** Validation study, Cancer, Comorbidity, Administrative data

## Abstract

**Background:**

Population-level health administrative datasets such as hospital discharge data are used increasingly to evaluate health services and outcomes of care. However information about the accuracy of Australian discharge data in identifying cancer, associated procedures and comorbidity is limited. The Admitted Patients Data Collection (APDC) is a census of inpatient hospital discharges in the state of New South Wales (NSW). Our aim was to assess the accuracy of the APDC in identifying upper gastro-intestinal (upper GI) cancer cases, procedures for associated curative resection and comorbidities at the time of admission compared to data abstracted from medical records (the ‘gold standard’).

**Methods:**

We reviewed the medical records of 240 patients with an incident upper GI cancer diagnosis derived from a clinical database in one NSW area health service from July 2006 to June 2007. Extracted case record data was matched to APDC discharge data to determine sensitivity, positive predictive value (PPV) and agreement between the two data sources (κ-coefficient).

**Results:**

The accuracy of the APDC diagnostic codes in identifying site-specific incident cancer ranged from 80-95% sensitivity. This was comparable to the accuracy of APDC procedure codes in identifying curative resection for upper GI cancer. PPV ranged from 42-80% for cancer diagnosis and 56-93% for curative surgery. Agreement between the data sources was >0.72 for most cancer diagnoses and curative resections. However, APDC discharge data was less accurate in reporting common comorbidities - for each condition, sensitivity ranged from 9-70%, whilst agreement ranged from κ = 0.64 for diabetes down to κ < 0.01 for gastro-oesophageal reflux disorder.

**Conclusions:**

Identifying incident cases of upper GI cancer and curative resection from hospital administrative data is satisfactory but under-ascertained. Linkage of multiple population-health datasets is advisable to maximise case ascertainment and minimise false-positives. Consideration must be given when utilising hospital discharge data alone for generating comorbidity indices, as disease burden at the time of admission is under-reported.

## Background

The assessment of health services utilisation and associated patient outcomes are fundamental to improving health care performance. However, traditional investigation methods such as clinical cohort investigations are resource-intensive and costly. Increasingly, population-level health administrative data, such as hospital discharge and registry data, used alone or linked with other datasets, are being used as a cost-effective and resource-efficient alternative to investigate population treatment patterns, health service utilisation and outcomes of care [1,2] across a range of conditions [1,3-9].

Analyses using health administrative data is generally based on the assumption that the data sets have high levels of accuracy in identifying medical conditions and associated treatments and services. In particular there is widespread use of hospital discharge data in this context, yet there are relatively few published studies reporting the accuracy of hospital discharge diagnostic and procedure codes. Some of the well documented limitations are missing data, abstraction errors and misclassification errors [10]. Therefore investigations using these data requires high level expertise from the perspective of the analysts and in the interpretation of findings [11,12].

In Australia, there have been series of validation studies investigating the accuracy of hospital discharge data in identifying obstetric conditions and outcomes [13-16] but there are fewer studies examining the accuracy of cancer related diagnoses [17-19] and treatment in discharge data [7,20]. Clearly the identification of accurate population level cancer-related measures from hospital discharge data is essential to improved understanding of treatment processes and outcomes of care. This is particularly important in circumstances where discharge data is used as the only information source and not linked to other population datasets such as cancer notifications.

Upper gastro-intestinal (upper GI) cancers account for 7% of all incident cancers and 15% of all cancer deaths in Australia [21]. Surgical resection for curable upper GI cancers is the standard treatment, with or without adjuvant chemotherapy. We previously reported on patient outcomes following curable surgical resection for oesophageal cancer in New South Wales (NSW), the largest jurisdiction in Australia, using linked administrative health data [5]. We expand on this work by examining the accuracy of hospital administrative discharge diagnostic codes in identifying site-specific upper GI cancer cases and procedure codes for those undergoing curative resection for site-specific cancer. We also examine the accuracy of specific comorbidities as recorded in hospital discharge data compared with those listed in patient medical records.

## Methods

### Setting

Australia has a publicly-funded universal health care system. All Australian citizens and permanent residents are entitled to subsidised treatment from medical practitioners and fully subsidised (free) treatment in public hospitals [22]. NSW is the largest jurisdiction in Australia, and until 2011 comprised eight area health services.

### Study population

Our study population was potential patients with data indicative of a primary incident upper GI cancer (International Classification of Diseases v10 [ICD-10] codes C15, C16, C22 and C25) in the period July 2006 to June 2007 in an area health service (AHS) clinical database. Cases were confirmed using data extracted from their medical records.

The South Eastern Sydney Illawarra Area Health Service commenced capture of the diagnostic and treatment details of all patients diagnosed with cancer after 1^st^ January 2006 or receiving part or all of their treatment within a health service facility. However, the AHS clinical database does not distinguish between primary and secondary cancer diagnoses. Nevertheless, this was the most systematic and cost-effective approach available to us for identifying potential upper GI incident cancer cases from which data could be extracted from patient medical records. Data extracted from the medical records of confirmed cases was considered to be ‘the gold standard’.

### Hospital discharge database

The Admitted Patient Data Collection (APDC) is a census of all inpatient separations (discharges) from all public, private and repatriation hospitals, private day procedures centres and public nursing homes in NSW. Hospital medical coders abstract data from patient medical records following discharge and submit details to the NSW Health Information Exchange for every episode of care. A separate record is processed for each period of inpatient care, irrespective of the time interval between the date of separation and subsequent readmission.

### Data linkage

**Figure 1:**
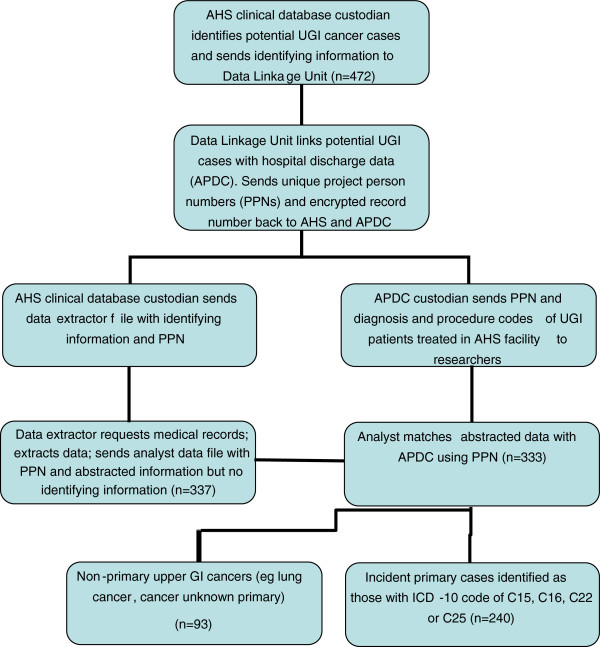
Linkage process

The data linkage process is shown in Figure 1. The AHS data manager extracted the relevant potential cases with patient identifiers (eg: name, medical record number, date-of-birth) and forwarded the extract to the Centre for Health Record Linkage (CHeReL). The CHeReL matched AHS cases to APDC records using probabilistic linkage and best privacy preserving protocols [23]. Each case and APDC record was assigned a unique identifier (or Project Person Number: PPN) so as to match individuals across the two data sets. The research team received two individual data files: patients with a diagnosis or treatment of upper GI cancer within an AHS facility as recorded in the APDC with PPNs but not patient identifiers were forwarded to the team analyst; and the AHS cases with PPNs and identifying information was sent to the data abstractor who used the information to identify medical records for review. Data were extracted from hospital records and sent to the research team analyst with PPNs and no other personal identifying data information. Using the PPN, the analyst merged the abstracted hospital record data with the APDC.

### Case record extraction

A data extraction form was developed and pilot tested by the research team in consultation with a medical registrar and gastroenterologist. The final version of the abstraction tool had 12 items regarding patient characteristics, cancer-specific diagnostic and treatment characteristics and the presence of common comorbidities (eg hypertension, diabetes, ischaemic heart disease) as suggested for inclusion by the consultants. Most of the items had response options whereby the trained extractor was required to indicate the presence of specific characteristics. To assess inter-rater reliability, a second trained researcher extracted data from a random selection of at least 10% of medical records (n = 38; 12%) independently. Both extractors were health care professionals trained in data abstraction by the medical registrar, and were blind to the diagnostic and treatment details of patients as described in the APDC.

### Statistical analyses

We calculated sensitivity and positive predictive value (PPV) of the APDC data against the case record data (gold standard) for the following: 1) diagnosis for site-specific upper GI cancer; 2) curative resection for site-specific cancer and 3) comorbid conditions. Sensitivity was calculated as the proportion of cases/procedures of cancer or comorbidity reported in the APDC as compared with the true diagnosis/procedure as determined by the case data. We did not calculate specificity or negative predictive value as the denominator (the population without a GI cancer diagnosis) was not ascertained.

Of the persons with site-specific cancer diagnosis/procedure or comorbid condition in the entire APDC (ie true and false report), the PPV was calculated as the proportion with a matching case/procedure from abstracted data (ie true report).

We determined the agreement between the case medical record data and the APDC using the kappa statistic, which adjusts for the agreement that would be observed on the basis of chance. A κ-value >0.75 is an indication of excellent agreement whilst that between 0.40 and 0.75 represents fair to good agreement [24].

We identified surgical resections in the APDC using the Medicare Benefits Schedule-Extended classification of the International Classification of Diseases (ICD_10_AM) procedural block codes for oesophagectomy (0858–0860), gastrectomy (875–879), pancreatectomy and excision of lesions of pancreas (978 and 979 90294–01, 30578–00) and excision procedures of the liver (953) [25]. Upper GI cancer classification recorded in the APDC as a reason for the episode of care, or comorbidities were identified using ICD_10_AM diagnostic codes from the primary and up to 10 secondary diagnostic fields. Comorbidity ICD-10 diagnostic codes were as follows: hypertension (I10-I15), diabetes (E10.1, E10.5, E10.9, E11.1, E11.5, E11.9, E13.1, E13.5, E13.9, E14.1, E14.5, E14.9), ischemic heart disease (I2-I25), GORD (K21.0, K21.9), alcohol abuse (F10.1, F10.2, K70, Z72.1, Z86.41), hepatitis B or C (B16, B18, B19, K73), chronic obstructive pulmonary disease (J44) and dementia (F01-F03).

We also used the kappa statistic to calculate inter-rater reliability of the extracted data for cancer diagnosis, curative resection and comorbidity. Inter-rater reliability between the record extractors was κ = 0.91 and κ = 0.74 for overall site-specific cancer diagnosis and curative surgery respectively. Agreement for specific comorbidities was κ =1.00 for diabetes, ischaemic heart disease and hypertension and κ =0.64 for gastro-oesophageal reflux disease (GORD). Hence we felt the data obtained by the main extractor could be analysed with confidence.

All statistical analyses were performed using SAS software, version 11.2 (SAS Institute Inc, Cary, NC).

The study was approved by the NSW Population and Health Services Research Ethics Committee (Ref 2010/05/253) and site specific approvals from Prince of Wales, St George, Sutherland and Wollongong Hospitals.

## Results

### Cohort characteristics

Of the 472 potential cases identified in AHS clinical database, 337 (71%) were available for review; however due to mismatching errors during the data linkage process, four records did not link to the APDC. Of the remaining 333 medical records 240 patients had an incident diagnosis of upper GI cancer; this constituted our study cohort. The majority of patients without incident upper GI cancer (n = 91/333) had another primary cancer type (eg lung, renal cell, unknown primary site) and there was insufficient information in the medical records or associated notes to determine cancer diagnosis type for the remaining two patients.

Over half of the 240 patients with upper GI cancer as classified in the medical chart review were >70 years of age in 2006 (55%), with 35% aged 51–70 years; comparable to patients with no primary diagnosis of upper GI cancer (63% and 27% respectively). 32% (n = 76) of the cases had an incident diagnosis of pancreatic cancer, 30% (n = 71) gastric cancer, 25% (n = 59) oesophageal cancer and 14% (n = 34) liver cancer. Only 27% (64/240) of all upper GI cancer patients had curative surgery; the majority being for gastric cancer (42%) and pancreatic cancer (27%). The most common comorbidity reported in the study cohort was hypertension (50% of patients), with fewer reports of diabetes (32%), GORD (20%), ischaemic heart disease (IHD: 18%), alcohol abuse (9%) or Hepatitis B or C (7%). Similar comorbidity profiles were found in the patients not classified with upper GI cancer from the medical chart review.

### Cancer diagnosis and surgical resection

Compared with the medical records, overall sensitivity for site-specific cancer diagnosis for the APDC was 89% (95% CI 84-93%). Sensitivity for each cancer diagnosis was satisfactory, ranging from 80% (95% CI 67-89%) for oesophageal cancer to 95% (95%CI 86-98%) for pancreatic cancer (Table 1). Overall sensitivity for curative surgery for upper GI cancer from the APDC was 84% (95%CI 73-92%), ranging from 67% (95% CI 24-94%) for oesophageal cancer to 91% (95%CI 57-99%) for liver cancer.

**Table 1:** Accuracy of upper GI diagnosis and curative resection reporting, plus reporting of comorbidities in APDC discharge data

	**Medical records -Condition present**	**True positive**	**Sensitivity (95% CI)**	**False positive**	**Positive predictive value**	**κ-coefficient**
**UGI cancer diagnosis**	240	214	89.2 (84.4-92.7)			
Oesophagus	59	47	79.7 (66.8-88.6)	18	72.3 (60.4-81.7)	0.72 (0.63-0.81)
Gastric	71	63	88.7 (78.5-94.7)	22	74.1 (63.9-82.2)	0.75 (0.62-0.89)
Liver	34	32	94.1 (79.0-99.0)	45	41.6 (31.8-52.3)	0.42 (0.30-0.52)
Pancreas	76	72	94.7 (86.4-98.3)	17	80.9 (71.5-87.7)	0.84 (0.78-0.90)
**Curative surgery**	64	54	84.4 (72.7-91.9)			
Oesophagus	6	4	66.7 (24.1-94.0)	1	80.0 (30.0-98.9)	0.79 (0.52-0.98)
Gastric	30	27	90.0 (72.3-97.4)	7	79.4 (63.2-89.6)	0.83 (0.74-0.94)
Liver	11	10	90.9 (57.1-99.5)	8	55.6 (33.7-75.4)	0.68 (0.48-0.87)
Pancreas	17	13	76.5 (49.8-91.2)	1	92.9 (68.5-98.7)	0.83 (0.69-0.98)
**Comorbid conditions**						
Hypertension	120	74	61.7 (52.3-70.3)	50	59.7 (50.9-67.9)	0.37 (0.28-0.47)
Diabetes	78	45	57.7 (46.0-68.6)	1	97.8 (87.0-99.9)	0.64 (0.53-0.74)
IHD*	42	20	47.6 (32.3-63.4)	17	54.0 (37.1-70.2)	0.41 (0.26-0.56)
GORD*	47	4	8.5 (2.8-21.3)	20	16.7 (5.5-38.2)	<0.01 (−−, 0.09)
Alcohol abuse	21	12	57.1 (34.4-77.4)	16	42.9 (26.5-60.9)	0.40 (0.22-0.60)
Hepatitis B or C	17	6	35.3 (15.3-61.4)	3	66.7 (35.4-87.9)	0.47 (0.22-0.73)
COPD*	7	3	42.9 (11.8-79.8)	8	27.3 (7.3-60.7)	0.33 (0.04-0.68)
Dementia	10	7	70.0 (35.4-91.9)	4	63.6 (35.4-84.8)	0.72 (0.51-0.96)

* IHD = Ischaemic heart disease;42 GORD = gastro-oesophageal reflux disease; COPD = chronic obstructive pulmonary disease.

Misclassification was the most common reason for false-negative reports in the hospital discharge data; for diagnosis 11 oesophageal cancers were classified as gastric cancer, whilst seven gastric, two liver and four pancreatic cancers were misclassified as another primary cancer (such as lung cancer or renal cell carcinoma). Two surgeries for oesophageal cancer were misclassified in the discharge data as occurring for gastric cancer, three pancreatic and three gastric cancer surgeries were misclassified as occurring for liver cancer and one liver cancer was assigned to another primary cancer (renal cell).

PPV for diagnosis of incident cancer in the APDC ranged from 42% for liver cancer to 81% for pancreatic cancer. With the exception of liver cancer, PPV for curative surgery was reasonably high (ranging from 79% to 93%).

Hence, although incident cases and procedures were under-ascertained, fair to very good agreement between the two data sources was shown for cancer diagnosis (κ-coefficient ranging from 0.42 to 0.84) and curative surgery (κ-coefficient ranging from 0.68 to 0.83) (Table 1).

### Comorbidity

Sensitivity was highest for dementia (70.0%, 95%CI 35-92%) and lowest for GORD (8.5%, 95%CI 3-21%). PPV was variable, ranging from 17% (95%CI 6-38%) for GORD to 98% (95%CI 87-100%) for diabetes. Agreement between the two data sources was low to fair. Comorbidities were underreported in the APDC.

## Discussion

There is widespread and increasing use of population-level hospital discharge diagnostic and procedural codes to monitor processes and outcome of care for health services research. The validation of coding in health administrative datasets has been identified as a priority in health services research by an international consortium [26]. However, validation studies remain uncommon [11]. This study contributes to the body of knowledge regarding the accuracy of administrative hospital discharge data (APDC) for cancer-related diagnoses, associated resection and comorbidity. This study also adds to the current literature on the nature and extent of reporting of comorbid diseases in population administrative data.

The distribution of site-specific cancers reported in our study was similar to the distribution of upper GI cancers in the Australian population [21]. The proportion of patients undergoing curative resection was also consistent with other studies [5,27,28]. We demonstrated that the APDC records cancer diagnosis and procedures at an acceptable level when compared to the medical record gold standard. We obtained similar levels of sensitivity for diagnosis compared to previous validation studies examining other cancers [17-19]. Previous validation studies examining breast and prostate cancer diagnoses have also shown surgical procedure records in administrative data to be well reported both in New South Wales [7,20] and internationally [29,30]. Nevertheless, true incident cancer cases and procedures from administrative data are still under-reported [31,32] and hence linkage with other datasets, such as registry-based data is recommended for case-ascertainment. Conversely, using hospital discharge data as the source for detecting incident upper GI cancer will over-estimate the incident rate, as false-positives are reported.

Our study also demonstrated the lower validity of common comorbidities reported in hospital discharge data. Despite an improvement in the accuracy of comorbid coding (with the introduction of ICD-10 coding) in administrative data over recent years, sensitivity when compared with medical records is generally low [33]. Our finding is consistent with a previous Australian study reporting under-ascertainment in more than 80 of 100 conditions in hospital discharge data [34]. The under-ascertainment of comorbidities in administrative data has been attributed to incompleteness of data transfer from medical records in individual hospitals to administrative databases [34-36]. Hospital coders are required to report medical conditions that affect the specific admission, however financial incentives may also impact on comorbidities which are reported. For example, recording of certain comorbidities over others may occur due to their effect on patient length-of-hospital-stay and procedures performed resulting in greater financial re-imbursement to the hospital. Misclassification of similarly related diseases (eg Barrett’s oesophagus versus GORD, COPD versus emphysema) may also occur. Clearly, the under-reporting of common comorbidities has implications for researchers using hospital discharge data as the sole source for assessing incidence, procedures, health outcomes and patient comorbidity as our study and others have identified that case ascertainment is likely to be incomplete.

Population dataset linkage is a cost-effective and attractive method for undertaking health services research, as large representative cohorts can be investigated efficiently when compared with traditional methods of recruiting participants from the population of interest. However, the validation of codes used to identify patients with particular health states and/or undergoing hospital procedures is essential to avoid misclassification bias which has the potential to undermine the internal validity and interpretation of study findings. This validation study showed that hospital discharge data has some limitation in the reporting of cancer, related curative resection and comorbidity. The outcomes from this validation study on cancer-related hospital administrative data will be important for consideration of future cancer surgical prevalence and outcomes by researchers and policy makers.

However, this validation study was limited to one clinical cancer group in an urban AHS in NSW, of which only 70% of the cases could be reviewed. Nevertheless, as cases were from four different hospital sites and the capture of cases is extremely high, it was thought that selection bias would be minimal and that the medical record data were indicative of records in the AHS clinical database. However, future studies should assess data accuracy across multiple jurisdictions and across several cancers. In addition, we used an AHS clinical database to assist in identifying potential GI cancer cases, as doing otherwise would have been significantly more time and cost intensive. This method does not allow for estimates of the NSW population without upper GI cancer, hence specificity and negative predictive value of the hospital discharge data could not be measured.

## Conclusions

Hospital administrative data provide a valid method of investigating health outcomes. However cases, procedures and comorbidity in population-level hospital discharge data is under-ascertained and hence researchers and policy-makers should acknowledge this in research and health planning assessments. Linkage across multiple datasets is recommended to improve case ascertainment.

## Competing interests

The authors declare that they have no competing interests.

## Authors’ contributions

EPS designed the study, drafted the manuscript, and conducted the statistical analyses. NP helped to draft the manuscript. SP helped design the study and to draft the manuscript. All authors read and approved the final manuscript.

### Funding

This study was funded by a Cancer Institute NSW Epidemiology Linkage Innovation Grant (ID Number: 10/EPI/2-01). Dr Pearson is funded as a Cancer Institute NSW Career Development Fellow (ID Number: 09/CDF/2-37).

## Pre-publication history

The pre-publication history for this paper can be accessed here:

http://www.biomedcentral.com/1472-6963/12/331/prepub
